# Development of a Novel Immunoprotective Culture System for Parathyroid Allografts: Utilizing Static Magnetic Fields to Modulate Lymphocyte Migration

**DOI:** 10.3390/cimb48040388

**Published:** 2026-04-10

**Authors:** Ahmed Alperen Tuncer, Gülnihal Bozdağ, Özge Karabıyık Acar, Fikrettin Şahin, Gamze Torun Köse, Erhan Ayşan

**Affiliations:** 1 Department of Genetics and Bioengineering, Faculty of Engineering and Natural Sciences, Yeditepe University, Istanbul 34755, Türkiye; alperen.tuncer@yeditepe.edu.tr (A.A.T.); gulnihal.bozdag@yeditepe.edu.tr (G.B.); fsahin@yeditepe.edu.tr (F.Ş.); gamzekose@yeditepe.edu.tr (G.T.K.); 2 Department of Genetics and Bioengineering, Istanbul Okan University, Istanbul 34959, Türkiye; ozge.acar@okan.edu.tr; 3 Department of General Surgery, Yeditepe University Hospital, Istanbul 34718, Türkiye

**Keywords:** parathyroid allotransplantation, static magnetic field (SMF), microencapsulation, immunomodulation, Jurkat cells, parathormone (PTH), biocompatibility, cell migration

## Abstract

Parathyroid allotransplantation is a promising treatment for hypoparathyroidism, yet immune rejection and fibrosis remain significant barriers. This study evaluates a novel immunoprotective culture system utilizing a moderate-intensity static magnetic field (SMF) to modulate lymphocyte migration without compromising graft functionality. Human parathyroid cells were encapsulated and divided into 10 experimental groups, co-cultured with Jurkat T-lymphocytes, and either exposed to SMF or maintained as controls. Over 72 h, we analyzed parathormone (PTH) secretion, cell viability (via proliferation assays), and molecular expression patterns of key markers (VitDR, PTH, GCM2, and CaSR). Lymphocyte dynamics were monitored through comparative imaging and cytokine profiling (IL-1α, IL-1β, and IL-2). SMF exposure significantly altered Jurkat cell behavior; while lymphocytes in unexposed groups aggregated around microcapsules, they were effectively repelled and migrated away from the graft interface under SMF exposure. Crucially, this biophysical manipulation was safe: no significant differences in PTH secretion or viability were observed across groups. All groups maintained essential genetic markers. Our findings demonstrate that SMF exposure induces lymphocyte migration away from the capsule without compromising parathyroid cell characteristics or functionality. Integrating encapsulation with SMF represents a novel, non-pharmacological, non-invasive immunoprotective strategy for parathyroid allotransplantation, offering a technological alternative to systemic immunosuppression.

## 1. Introduction

Parathyroid glands are small endocrine organs that secrete parathyroid hormone (PTH) when the circulating calcium level is reduced, which is important for the organism’s homeostasis [1,2,3,4]. PTH is crucial for a healthy balance of circulating calcium and phosphate levels [5,6,7]. Either excessive or deficient amounts of PTH define the state of parathyroid gland disorder. Hypoparathyroidism, characterized by insufficient PTH production, is a serious endocrine disorder, often resulting from iatrogenic damage during thyroid or parathyroid surgery [8,9]. Hypoparathyroidism can also be inherited and caused by immune system-related damage [9]. PTH is essential for calcium and phosphate homeostasis, and its deficiency leads to a chronic need for external calcium and Vitamin D supplementation or synthetic PTH therapy [10]. These conventional treatments are often associated with side effects, high costs, and reliability issues for long-term management [11].

Parathyroid allotransplantation is considered a promising, long-term therapeutic alternative [12]. However, similar to other allografts, success is severely limited by the host immune response, which necessitates the use of systemic immunosuppressants that carry significant side effects [13,14]. Tissue rejection involves both humoral and cellular immunity, with Natural Killer cells and T-lymphocyte activation being key contributors to cellular rejection [15]. To mitigate this challenge, advanced strategies focus on creating immune barriers or using external factors to modulate the immune response. For many years, hydrogels have been extensively used in tissue engineering due to their non-toxic, biodegradable, mucoadhesive, and viscoelastic characteristics and their ease of fabrication [16,17,18,19,20,21,22]. Cell and tissue encapsulation, typically using hydrogels like alginate, serves as a crucial technique. Alginate-based microencapsulation provides a selectively permeable, biocompatible matrix that allows for nutrient exchange while potentially shielding the graft from larger immune components, thus reducing the reliance on post-transplantation immunosuppressive drugs [23,24,25,26]. Despite the unique properties of alginate encapsulation, the immune response remains a significant barrier. Therefore, additional non-invasive immune suppression techniques are sought. It has been previously shown that lymphocytes actively migrate toward the cathode in an applied direct electric current (DC) field [27]. Harnessing this electrokinetic phenomenon to repel immune cells from the transplant site is a viable approach. However, the clinical implementation of a continuous electrical field for an in vivo graft is highly challenging due to the need for a constant power source and implanted wires, posing significant technical and bioethical hurdles. To circumvent these limitations, we propose the use of a static magnetic field (SMF) as a non-invasive alternative to the electric field. According to Ampère’s force law and the relationship between electricity and magnetism, a magnetic field can exert forces on moving charges. The magnetic field is a physical quantity that describes the influence of magnetism on moving electric charges, electric currents, and magnetic materials. It is quantitatively expressed in tesla (T) in the International System of Units (SI), or in gauss (G) in the CGS system, where 1 T = 10,000 G [28]. Magnetic fields are categorized into four types based on magnetic induction intensity, direction, and source: static magnetic field, pulsed magnetic field, rotating magnetic field, and alternating magnetic field. SMFs are a fundamental and highly utilized form of magnetic energy in biological studies [29]. According to magnetic induction intensity, SMFs can be classified as hypomagnetic fields (<5 μT), weak magnetic fields (5 μT–1 mT), moderate magnetic fields (1 mT–1 T), and high magnetic fields (>1 T). The International Commission on Non-Ionizing Radiation Protection (ICNIRP) has established that the magnetic induction intensity in the head and torso for occupational exposure should not exceed 2 T; since there are no large blood vessels or organs in the extremities, magnetic induction intensities below 8 T are considered safe [28,29]. Previous studies have explored the effects of magnetic fields on various cells in vitro [30,31], though specific investigations into parathyroid cell behavior under SMF influence, or its application in modulating immune response for transplantation, are lacking [32,33,34]. The biological influence of SMFs is mediated through several established molecular mechanisms. Moderate-intensity SMFs have been shown to influence reactive oxygen species (ROS) levels [35,36,37,38] and calcium (Ca^2+^) signaling dynamics [39,40,41,42], often in an intensity- and cell-type-dependent manner. Furthermore, SMFs can induce the orientation of diamagnetic biological molecules, such as actin and tubulin, leading to significant cytoskeletal reorganization [43,44,45] While many studies focus on the inhibitory or toxic effects of high-intensity fields, the potential to leverage these biophysical interactions—specifically for the directed spatial modulation of immune cells—remains an underexplored frontier in transplantation bioengineering [46,47,48,49].

This work evaluates the combined effect of alginate-based cell encapsulation and SMF application to enhance the success rate of parathyroid allotransplantation in vitro without immunosuppressants. By protecting the cells with alginate and utilizing the SMF to force the migration of lymphocytes (Jurkat cells) away from the capsule, we aim to demonstrate a potential immune response-free method that preserves the full functionality of the transplanted parathyroid cells, paving the way for a long-term therapeutic solution for hypoparathyroidism.

## 2. Materials and Methods

### 2.1. Cell Isolation from Parathyroid Tissues

Human parathyroid tissues were obtained from patients who underwent parathyroidectomy for primary hyperparathyroidism. Before the operations, informed consent from the patients and the approval of the local human ethics committee were obtained. This study strictly adhered to the principles of the Declaration of Helsinki. Approval was granted by the Bezmialem Vakıf University Clinical Research Ethics Committee of the Republic of Türkiye for human trials (2013/99) (Clinical ID: NCT02134483). Four patients with similar anamneses and indications, all diagnosed with primary hyperparathyroidism, were scheduled for parathyroidectomy, and the clinical characteristics of these donors are summarized (Table 1). The excised tissues were first examined histopathologically in the pathology laboratory, where a diagnosis of parathyroid hyperplasia was confirmed. Tissues were transferred to the laboratory in harvest medium (Dulbecco’s Modified Eagle Medium (DMEM), including 1000 units/mL of penicillin/streptomycin (P/S)).

After removing connective and adipose tissues in cold 1X phosphate-buffered saline (PBS), the parathyroid tissue was minced. PBS was removed, and collagenase type II (1 mg/mL), DNase-I (0.05 mg/mL), and bovine serum albumin (BSA, 5 mg/mL) were added to the minced tissues. The addition of DNase-I was crucial to decrease solution viscosity and prevent cell aggregation by breaking down extracellular DNA. Tissue solution (including pieces) was taken to a sterile 50 mL Falcon tube and transferred into a shaking 80 revolutions per minute (rpm) water bath for 30 min at 37 °C. Every 10 min, mechanical fragmentation was taking place with serological pipettes. After that, tissue pieces were settled down via centrifugation for 5 min at 500 rpm. Supernatant was filtered through a 40 µm cell strainer, and the remaining volume was filled up to approximately 40 mL by using sterile growth medium, which contains DMEM/Ham’s F12 medium supplemented with 1X ITS+Premix containing insulin (5 µg/mL), transferrin (5 µg/mL), selenious acid (5 ng/mL), and 100 U/mL of P/S. Filtered supernatant was centrifuged at 2000 rpm for 5 min. This procedure was repeated three times until the entire parathyroid tissue disappeared. The cell pellet was resuspended using growth medium. Parathyroid cells were counted using an automated cell counting device (Countess^®^ II FL, Thermo Fisher Scientific Inc., Bothell, WA, USA).

### 2.2. Study Design

For all groups, cell viability, cell proliferation, quantitative PTH secretion levels (biochemically assessed), and the expression levels of VitDR, PTH, GCM2, and CaSR genes by polymerase chain reaction (PCR) were evaluated at 24 and 72 h. In groups containing both lymphocytes and parathyroid cells (groups 5–10), IL-1α and IL-1β gene expression levels were also examined. All experiments were conducted in triplicate, with each experiment using 100,000 parathyroid cells per bead/well and 100,000 lymphocytes per well. For all groups, real-time images and videos were taken at 24 and 72 h to observe the movement characteristics of Jurkat cells. This study was conducted on the 10 groups (Table 2), and to ensure biological relevance and reproducibility, the study utilized four independent biological replicates (N = 4), with each replicate representing tissues derived from a distinct donor (Patients 1–4). For every biological sample, experimental procedures were performed in technical triplicate (n = 3), providing a total of 12 measurements per group. Real-time imaging and videos were captured at 24 and 72 h to evaluate the spatial dynamics of Jurkat cells.

### 2.3. Parathyroid and Jurkat Cell Cultures

Parathyroid cells were maintained in DMEM/Ham’s F12 complete medium. The medium was changed twice weekly. Cells were harvested using 0.25% trypsin-EDTA (1X, Gibco, Waltham, MA, USA) prior to the encapsulation process. The Jurkat cell line (ATCC, #TIB-152TM) served as the lymphocyte model. Jurkat cells were cultured in complete RPMI-1640 (1X, Gibco, Waltham, MA, USA) medium supplemented with 10% (*v*/*v*) FBS and 1% (*v*/*v*) P/S. The medium was changed twice weekly.

### 2.4. Calcium Alginate Bead Preparation

Pronova UP LVG Sodium alginate (Novamatrix, Sandvika, Norway, M/G ratio ≥ 1.5, Lot: BP-1905-07) and calcium chloride solutions (Multicell, Wisent Inc., Saint-Jean-Baptiste, QC, Canada) were prepared by dissolving the required amounts (for alginate solution 3% and calcium chloride 2% *w*/*v*) individually in Milli-Q water (Millipore, Burlington, MA, USA) under gentle agitation by constant stirring overnight. Based on the 3% (*w*/*v*) concentration and the high-G content of the Pronova UP LVG alginate used, the resulting matrix was characterized by a pore size of approximately 5–15 nm, facilitating the diffusion of PTH while maintaining immunoisolation properties. The sterilization of the solutions was carried out by filtering via polyvinylidene fluoride (PVDF) membrane (Millipore, Burlington, MA, USA) with a pore size of 0.22 µm inside the laminar flow hood.

### 2.5. Parathyroid Cell Encapsulation

Isolated parathyroid cells were gently mixed with a sterilized 3% (*w*/*v*) sodium alginate solution. This mixture was extruded dropwise using sterile 21-G syringes into a gelling bath of sterilized Calcium Chloride (CaCl_2_) cross-linker solutions to form cell-encapsulated beads. The resulting beads exhibited a mean diameter of 2598 ± 172 µm, consistent with our previously established and published standardized encapsulation protocol. The alginate beads were washed with Dulbecco’s Modified Eagle Medium–High Glucose (DMEM-HG) using sterile meshes to remove excessive divalent cations. A total of 5 min of incubation was applied individually in both gelation and washing solutions. After that, beads were transferred into 48-well plates containing complete DMEM/Ham’s F12. Empty beads were prepared using the identical protocol without cell addition to serve as controls.

### 2.6. Experimental Setup and Gauss/Tesla Meter Measurements

**Figure 1:**
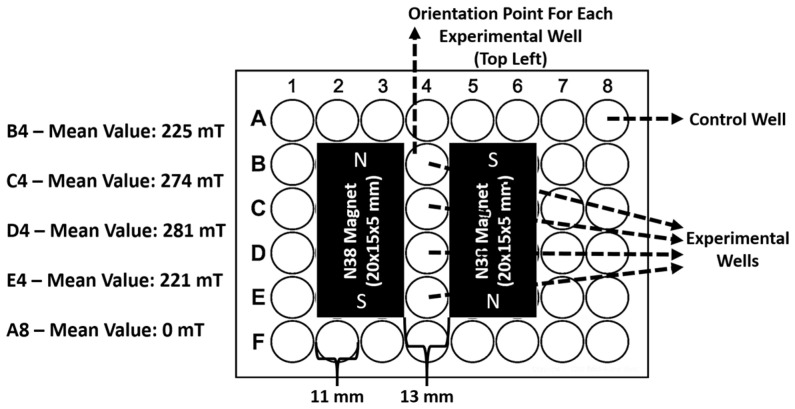
Detailed schematic and characterization of the static magnetic field (SMF) experimental setup for groups 2, 4, 6, 8, and 10. A 48-well plate was modified to integrate two N38 neodymium magnets (20 × 15 × 5 mm) positioned in an attracting North–South (N-S) orientation on opposite sides of the experimental wells. The magnets were placed with a side-to-side distance of approximately 13 mm, ensuring immediate proximity to the 11 mm diameter experimental wells in Column 4. Magnetic induction intensity was measured using a 5170 Gauss/Tesla Meter (F.W. BELL) across three independent replicates under both dry and wet (complete culture medium) conditions to ensure stability. The mean field intensity values for the experimental column were determined as B4: 225 mT, C4: 274 mT, D4: 281 mT, and E4: 221 mT. These measurements demonstrate a non-uniform field distribution, establishing a functional magnetic gradient within the experimental zone. Control wells (e.g., A8) were confirmed to have a mean value of 0 mT, ensuring isolation from the magnetic source and verifying that the magnetic flux density dissipated to negligible levels at this distance, thereby precluding any magnetophoretic influence on the control populations.

Experiments were conducted in 48-well plates (Corning, Corning, NY, USA). To integrate the magnetic source, specific regions of the plates were cut using a soldering gun (Heifer, JS98-A, Little Rock, AR, USA) and fitted with 20 × 15 × 5 mm N38 neodymium magnets arranged in an attracting North–South (N-S) orientation on opposite sides of the experimental wells. The 11 mm diameter wells were centered between the magnet faces, resulting in a side-to-side magnet distance of approximately 13 mm. The resulting static magnetic field (SMF) intensity was measured between the magnet poles using a 5170 Gauss/Tesla Meter (F.W. BELL, Orlando, FL, USA). Based on repeated measurements performed under both dry and wet conditions (in complete DMEM/Ham’s F12 and complete RPMI-1640 medium), the field intensity within the experimental zone ranged from 221 to 281 mT, establishing a functional magnetic gradient that facilitated directed cell migration. Control wells (Column 8) were confirmed at 0 mT, ensuring no magnetic exposure. Reproducible analysis of 10 experimental trials confirmed a consistent net directional vector of Jurkat cell aggregation toward the magnetic poles (Figure 1).

### 2.7. Cell Viability and Proliferation

Cell viability was assessed at 24 and 72 h for all experimental groups. Trypan Blue Staining (Invitrogen, Waltham, MA, USA, Lot#: 2303495) was performed by mixing 10 µL of the cell suspension (parathyroid or Jurkat) with 10 µL of stain in a 1:1 ratio. A total of 10 µL of the mixture was loaded onto a Countess Cell Counting Chamber Slide (Invitrogen, USA, Lot#: I45B0). The cell viability percentage and cell number were calculated and recorded using the Countess II FL Automated Cell Counter (Life Technologies, Carlsbad, CA, USA).

The MTS assay was performed on all groups at 24 and 72 h to assess comparative proliferation. The MTS Solution (CellTiter 96, Promega, Madison, WI, USA) was prepared in Basal RPMI-1640 (1X) medium at a 1:6 ratio under sterile, dark conditions. Cells in the 48-well plate received 600 µL of the MTS mixture. Incubation times at 37 °C with 5% CO_2_ differed by cell type: Jurkat cells were incubated for 4.5 h, and parathyroid cells for 2 h. Following incubation, 200 µL from each well was transferred to a 96-well plate and covered with aluminum foil. Absorbance values were measured at 490 nm using a spectrophotometer (Thermo Scientific, Varioskan Lux, Waltham, MA, USA) for both the calibration curve and experimental samples.

Encapsulated parathyroid cells, non-encapsulated parathyroid cells, and Jurkat cells were plated in a 48-well plate (Corning, USA) and incubated for 24 and 72 h ± SMF. Samples were washed with 1X PBS. The Live/Dead assay was performed by incubating the samples for 30 min at 37 °C with 5% CO2, using calcein-AM (live cell stain) and ethidium homodimer-1 (EtHD-1) (dead cell stain) (Invitrogen, USA). After a final wash with 1X PBS, samples were visualized using the fluorescent mode of an inverted fluorescent microscope (ZEISS Vert. A1, Jena, Germany). Cell viability percentages were calculated using ImageJ software (version 1.54).

### 2.8. Gene Expression Profile

Total RNA was isolated after 24 and 72 h using the Nucleospin RNA XS Kit Machery-Nagel, #740902.50, (MACHEREY-NAGEL GmbH & Co. KG, Düren, Germany). RNA and subsequent cDNA (synthesis performed using the iScript cDNA Synthesis Kit, Bio-Rad^®^, Hercules, CA, USA) purity were monitored via NanoDrop spectrophotometer (Thermo Scientific^®^). Gene expression was investigated for parathyroid markers (PTH, CaSR, VitDR, and GCM2) and Jurkat markers (IL-1α, IL-1β, and IL-2). Forward and reverse primers were designed for each gene (Table 3). PCR was carried out using iTaq Universal SYBR Green Supermix (2X) (Bio-Rad^®^). The β-actin gene was used for normalization and internal control purposes.

### 2.9. PTH Measurement

Conditioned medium was collected at 24 and 72 h and stored at −80°C. Prior to PTH measurement, thawed medium was centrifuged at 10,000× *g* for 10 min, and the supernatants were used. Secreted PTH concentration (pg/mL) was detected using a human PTH ELISA kit (Abcam, Cambridge, UK, ab230931) according to the manufacturer’s protocol. All samples were measured in triplicate. Blank medium values were individually subtracted from all measurements.

### 2.10. Live-Cell Imaging

For the magnetic field exposure experiments, Jurkat cells (100,000 cells/well) and encapsulated parathyroid cells (100,000 cells/bead) were seeded in the custom-prepared 48-well plates (with magnets positioned on either side). The plates were placed in a Live-Cell Imaging System (ZEISS-Axio Observer Z1, Jena, Germany) maintained at 37 °C and 5% CO_2_. Observation was performed for 72 h. Bright-field images of the entire well, containing encapsulated parathyroid cells, non-encapsulated parathyroid cells, and Jurkat cells, were taken at 24 and 72 h to monitor the effect of the magnetic field on the orientation and spatial distribution of Jurkat cells.

### 2.11. Statistical Analysis

Data analysis was conducted using GraphPad Prism 9 software (GraphPad Software, San Diego, CA, USA) (Version 9.0.2). Statistical differences were evaluated via two-way ANOVA followed by Tukey’s post hoc test for significant results (*p* < 0.05). Statistical significance levels were defined as a *p* < 0.0001, *p* < 0.001, *p* < 0.01, and *p* < 0.05. To ensure maximum legibility of the multi-group datasets, significance markers (asterisks) were omitted from the graphical representations. Instead, all primary comparisons—specifically the longitudinal changes between the 24 h and 72 h time points within each group—are reported as statistically significant (*p* < 0.05) and are described in detail within Section 3. Pairwise comparisons between different treatment groups at static time points were also evaluated, with only those reaching the *p* < 0.05 threshold being explicitly highlighted in the text. All results are expressed as the mean ± standard deviation (SD) based on the four independent biological replicates (N = 4), ensuring that the sample size for each group effectively accounted for donor-to-donor variability (n ≥ 12 total observations).

## 3. Results

In the present study, isolated parathyroid cells were prepared in both encapsulated (groups 3, 4, 9, and 10) and non-encapsulated (groups 1, 2, 7, and 8) forms. Some of the encapsulated or non-encapsulated parathyroid cells were also co-cultured with Jurkat cells (groups 7, 8, 9, and 10), and the effect of a moderate-intensity SMF on all groups (groups 2, 4, 6, 8, and 10) was investigated. At 24 and 72 h, PTH secretion, cell viability, and proliferation of all groups were assessed, along with the expression of parathyroid cell-specific (VitDR, PTH, GCM2, and CaSR) and Jurkat cell-specific genes (IL-1α, IL-1β, and IL-2). Images were also taken simultaneously with the moderate-intensity SMF at 24 and 72 h to conduct a comparative in-group analysis of Jurkat cell migration.

### 3.1. Cell Viability and Proliferation

**Figure 2:**
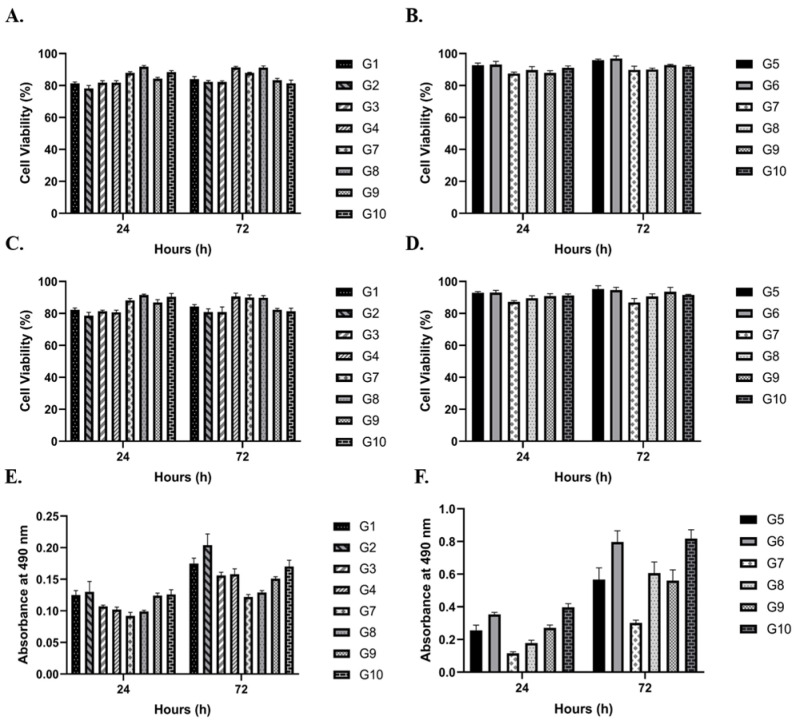
Longitudinal Assessment of Parathyroid and Jurkat Cell Viability and Proliferative Dynamics. (**A**–**D**) Comparative viability profiles and (**E**,**F**) metabolic activity/proliferation analysis of human parathyroid cells (**A**,**C**,**E**) and Jurkat T-lymphocytes (**B**,**D**,**F**) across 24 h and 72 h incubation intervals. Quantification was performed via trypan blue exclusion (**A**,**B**), fluorescence-based live/dead assays (**C**,**D**), and MTS metabolic activity assays (**E**,**F**). The initial cell density was standardized at 1 × 10^5^ cells per well/microcapsule. Data are expressed as mean ± SD derived from four independent biological replicates (N = 4) with technical triplicates (n = 12 total observations). To maintain graphical legibility and prevent data obstruction in multi-group comparisons, significance markers were omitted from the plots. Longitudinal analysis confirmed significant temporal shifts in parathyroid viability (Trypan: *p* < 0.01), Jurkat viability (Trypan: *p* < 0.0001; Live/Dead: *p* < 0.05), and metabolic activity for both cell types (*p* < 0.0001). Parathyroid fluorescence-based viability (**C**) exhibited no significant temporal variation (*p* > 0.05).

In both the encapsulated and non-encapsulated groups (groups 1–10), there was no difference in cell viability and proliferation values for parathyroid and lymphocyte cells between 24 h and 72 h. The mean cell viability for parathyroid cells, assessed using Trypan blue staining, was 84.6% at 24 h and 85.5% at 72 h (Figure 2A) (*p* < 0.01). For Jurkat cells, the mean viability was 90.3% at 24 h and 92.8% at 72 h, regardless of the application of a moderate-intensity SMF (Figure 2B) (*p* < 0.0001). Similarly, the live/dead assay results showed comparable viability percentages to those observed with Trypan blue staining. According to the live/dead assay, the mean cell viability for parathyroid cells was 84.9% at 24 h and 85.7% at 72 h (Figure 2C) (*p* > 0.05). Jurkat cells also exhibited similar viability percentages in the live/dead assay, with 89.9% at 24 h and 92.2% at 72 h (Figure 2D) (*p* < 0.05). On the other hand, the MTS proliferation assay results indicated that the mean optical density values at 490 nm (OD490) for parathyroid cells were similar regardless of moderate-intensity SMF application at 24 and 72 h (0.113 and 0.158, respectively) (Figure 2E) (*p* < 0.0001). In contrast, the OD490 values for Jurkat cells were lower at 24 h than at 72 h (0.608 and 1.261, respectively) (Figure 2F) (*p* < 0.0001). However, both groups exhibited a similar increase for Jurkat cells, regardless of the application of a moderate-intensity SMF.

### 3.2. Gene Expression Profile

**Figure 3:**
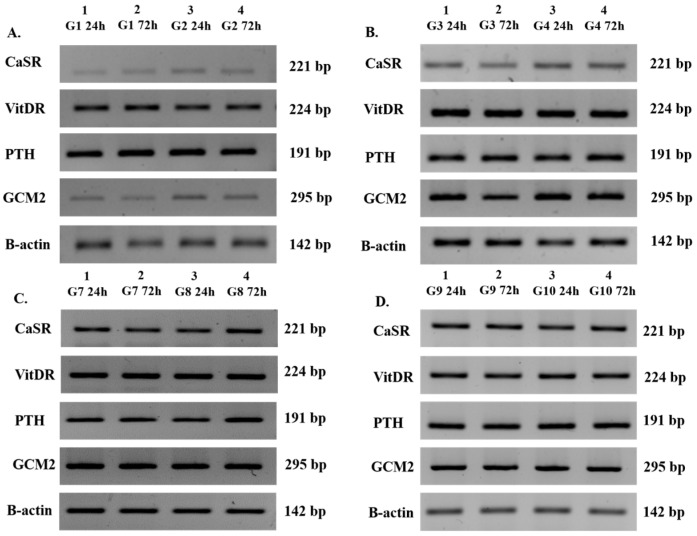
Qualitative Transcriptional Profiling of Parathyroid-Specific Biomarkers via Semi-Quantitative RT-PCR. (**A**–**D**) Electrophoretic analysis demonstrating the steady-state gene expression of CaSR, VitDR, PTH, and GCM2 in human parathyroid cell populations across experimental cohorts: (**A**) groups 1–2 (non-encapsulated controls), (**B**) groups 3–4 (encapsulated controls), (**C**) groups 7–8 (non-encapsulated co-cultures), and (**D**) groups 9–10 (encapsulated co-cultures) at 24 and 72 h post-incubation. β-actin was utilized as the internal constitutive loading control. The numerical annotations (e.g., 221 bp, 224 bp) denote the expected PCR amplicon lengths in base pairs (bp), serving as a molecular weight verification for the specificity of the amplified cDNA sequences. Bands consistently aligned with predicted nucleotide lengths for PTH (191 bp), CaSR (221 bp), VitDR (224 bp), and GCM2 (295 bp), confirming target-specific gene amplification across all parathyroid-containing groups.

**Figure 4:**
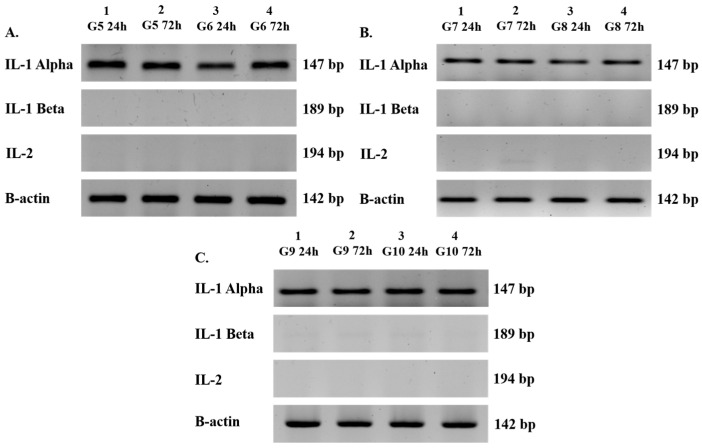
Qualitative Transcriptional Profiling of Jurkat T-Lymphocyte-Specific Biomarkers via Semi-Quantitative RT-PCR. (**A**–**C**) Electrophoretic analysis of IL-1α, IL-1β, and IL-2 genes in Jurkat cell populations at 24 and 72 h for (**A**) groups 5–6 (lymphocytes only), (**B**) groups 7–8 (non-encapsulated co-culture), and (**C**) groups 9–10 (encapsulated co-culture). Molecular sizes are expressed in base pairs (bp), representing the specific PCR amplicon lengths for each primer set. All visible bands matched the predicted nucleotide lengths for IL-1α (147 bp) and β-actin (142 bp), confirming target-specific gene amplification across all Jurkat-containing groups.

**Figure 5:**
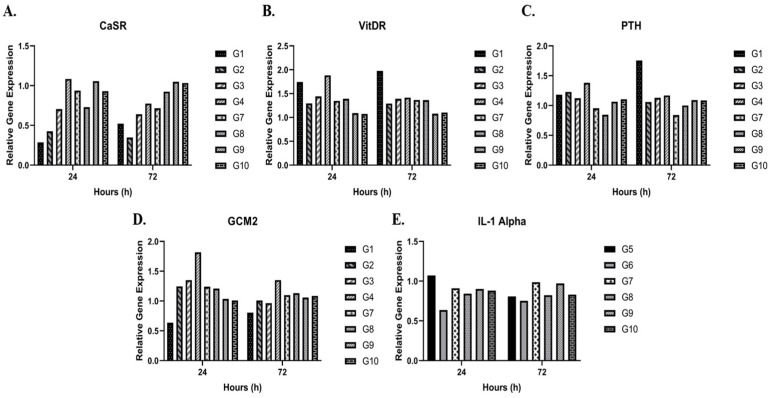
Comparative Transcriptional Analysis and Gene Expression Profiles of Parathyroid and Jurkat Cell Populations. (**A**–**D**) Longitudinal evaluation of relative gene expression levels for parathyroid-specific functional markers, including Calcium-Sensing Receptor (CaSR, (**A**)), Vitamin D Receptor (VitDR, (**B**)), Parathyroid Hormone (PTH, (**C**)), and Glial Cells Missing Homolog 2 (GCM2, (**D**)). (**E**) Relative expression of the pro-inflammatory cytokine IL-1α in Jurkat T-lymphocytes. Gene expression was quantified via RT-qPCR using the 2^−ΔΔCT^ method and normalized to the baseline at 24 and 72 h under moderate-intensity static magnetic field (SMF) exposure vs. control conditions. Data are represented as the mean fold change ± SD derived from four independent biological replicates (N = 4) with technical triplicates (n = 12 total observations). For optimal graphical clarity and to maintain the focus on longitudinal trends, significance brackets were omitted from the plots. Two-way ANOVA confirmed significant temporal shifts in CaSR (*p* < 0.05), VitDR (*p* < 0.01), and GCM2 (*p* < 0.05) expression. No significant temporal variation was observed for PTH (**C**) or Jurkat-derived IL-1α (**E**) transcripts (*p* > 0.05).

All measured groups (excluding groups 5 and 6) showed positive expression of the key parathyroid-specific genes: PTH (191 bp), CaSR (221 bp), VitDR (224 bp), and GCM2 (295 bp) at both 24 and 72 h (Figure 3A–D). Similarly, groups containing Jurkat cells (groups 7–10) were positive for IL-1α (147 bp) and β-actin (142 bp), while IL-1β (189 bp) and IL-2 (194 bp) were consistently negative at both time points (Figure 4A–C). The mean relative gene expression fold changes for all parathyroid markers (CaSR, GCM2, VitDR, and PTH) between 24 and 72 h remained consistent across all parathyroid cell-containing groups. The fold changes were 0.77 and 0.75 for CaSR (*p* < 0.05), 1.19 and 1.06 for GCM2 (*p* < 0.05), 1.41 and 1.37 for VitDR (*p* < 0.01), 1.11 and 1.14 for PTH (*p* > 0.05), respectively. These expression levels were not significantly affected by the application of a moderate-intensity SMF (Figure 5A–D). Consistently, in all Jurkat-containing groups (groups 5, 6, 7, 8, 9, and 10), the relative expression of IL-1α remained similar (0.87 ± 0.84 fold change, *p* > 0.05) over time, regardless of magnetic field exposure (Figure 5E). Overall, the PCR results demonstrate that the magnetic field had no impact on the baseline gene expression profiles of either cell type throughout the experiment.

### 3.3. PTH Measurement

**Figure 6:**
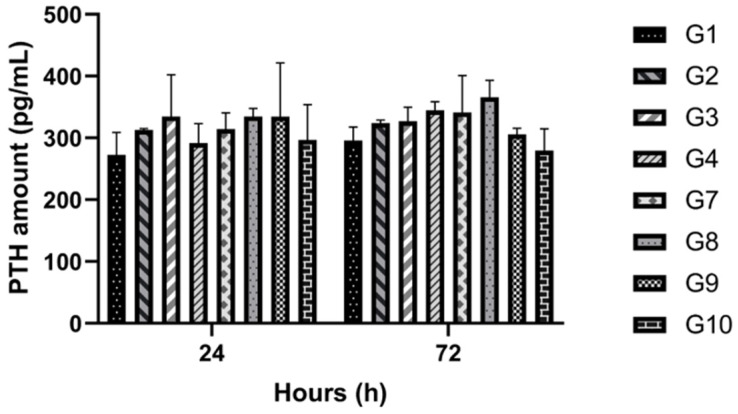
Quantitative Biochemical Evaluation of Parathyroid Hormone (PTH) Secretory Profiles. Comparative analysis of cumulative PTH secretion (pg/mL) in conditioned media across all parathyroid cell-containing experimental cohorts at 24 and 72 h post-incubation. Data are represented as mean values ± SD derived from four independent biological replicates (N = 4) with technical triplicates (n = 12 total observations). Statistical Analysis: Two-way ANOVA followed by Tukey’s post hoc test confirmed that moderate-intensity SMF exposure yielded no statistically significant deviation in hormone secretion compared to non-exposed controls (*p* > 0.05). The preservation of stable PTH levels (Mean 24 h: 311.45 pg/mL; Mean 72 h: 322.84 pg/mL) across both encapsulated and non-encapsulated formats validates the biophysical safety and functional biocompatibility of the SMF-integrated culture system for parathyroid allotransplantation.

PTH concentration (pg/mL) in the conditioned media was evaluated at 24 and 72 h (Figure 6). While groups exposed to the SMF (groups 2, 4, 8, and 10) generally showed slightly higher PTH levels compared to non-exposed control groups (groups 1, 3, 7, and 9) at 72 h, these differences were not statistically significant (*p* > 0.05). The overall mean PTH values for all parathyroid-containing groups remained consistent, measuring 311.45 pg/mL at 24 h and 322.84 pg/mL at 72 h, irrespective of SMF application. This confirms that the moderate-intensity magnetic field did not alter PTH secretion, supporting its safety and compatibility with the function required for successful parathyroid allotransplantation.

### 3.4. Live-Cell Imaging

**Figure 7:**
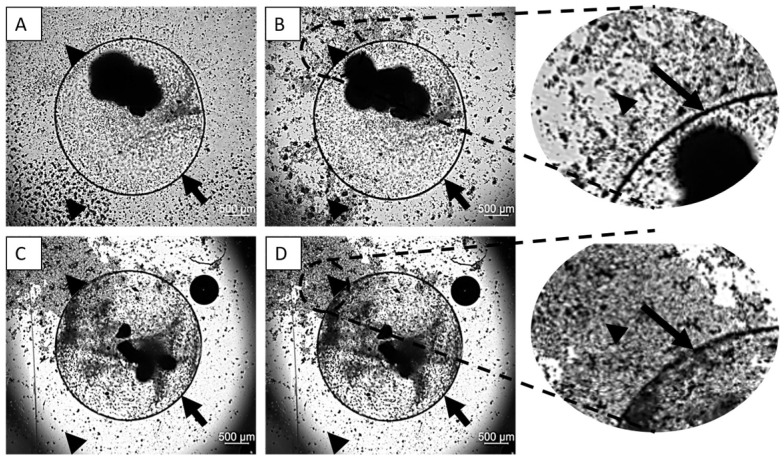
Spatiotemporal Analysis of Jurkat Cell Migration Dynamics via Live-Cell Imaging. (**A**,**B**) Representative bright-field micrographs of Group 9 (encapsulated parathyroid cells co-cultured with Jurkat lymphocytes in the absence of SMF) at 24 (**A**,**C**) and 72 (**B**,**D**) h, illustrating a randomized lymphocyte distribution at the capsule interface. (**C**,**D**) Corresponding images of Group 10 (SMF-exposed) demonstrating directional magnetophoretic migration and the emergence of distinct lymphocyte-depleted zones surrounding the alginate capsule. Magnification: 2.5×. Black arrows denote the sodium alginate microcapsule boundary; black arrowheads indicate Jurkat cell populations. Note: Dynamic mobilization and altered spatial distribution vectors are further documented in Supplementary Videos S1 and S2.

**Figure 8:**
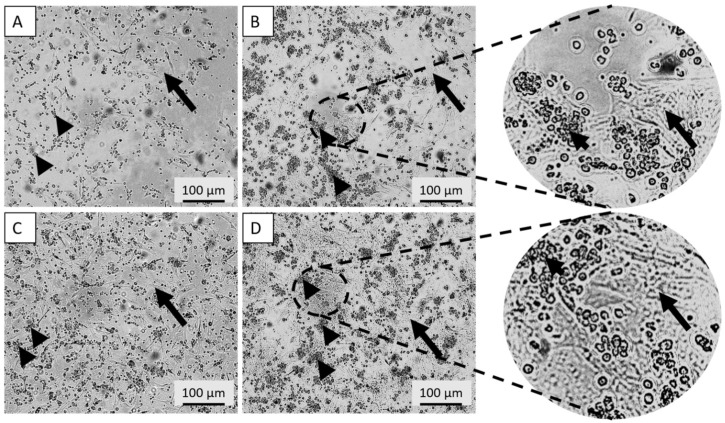
Comparative Evaluation of Lymphocyte Positioning in Non-Encapsulated Co-Culture Systems. (**A**,**B**) Micrographs of Group 7 (non-encapsulated parathyroid cells + Jurkat cells; SMF-) and (**C**,**D**) Group 8 (non-encapsulated parathyroid cells + Jurkat cells; SMF+) after 24 (**A**,**C**) and 72 (**B**,**D**) hours of incubation. In the absence of an alginate physical barrier, no significant directional migration or separation was observed regardless of magnetic field application, highlighting the essential synergistic role of the combinatorial encapsulation–SMF approach for immune cell modulation. Magnification: 10×. Arrows indicate non-encapsulated parathyroid cell clusters; arrowheads denote Jurkat cell distribution.

Live-cell imaging revealed distinct lymphocyte migration patterns over 72 h, dependent on both encapsulation and the SMF. Encapsulated groups (groups 9 and 10): In the absence of an SMF (Group 9), lymphocytes showed no significant migration pattern around the capsule (Supplementary Video S1A–C). However, in the presence of the SMF (Group 10), lymphocytes were observed to move consistently away from the encapsulated parathyroid cells (Supplementary Video S2A–C) (Figure 7A–D). Non-encapsulated groups (groups 7 and 8): Lymphocytes co-cultured with non-encapsulated parathyroid cells exhibited no apparent migration pattern across the culture area at either 24 or 72 h, regardless of the application of the magnetic field (Figure 8A–D). These findings demonstrate that the SMF successfully induces spatial separation of immunocompetent cells; however, this effect is specifically observable when the allograft is protected by alginate microencapsulation, suggesting a combinatorial immunoprotective effect.

## 4. Discussion

Magnetic fields (MFs) are unique physical phenomena that can penetrate most materials, distinguishing them from classical mechanics. The biological impact of MFs has long been investigated, particularly their influence on cellular and molecular functions [28,29]. Clinically, magnetic resonance imaging (MRI), introduced in the early 1980s, exposes patients to strong MFs yet remains widely accepted as safe, with virtually no adverse effects reported [30]. This safety stems from the low magnetic susceptibility of human tissues and the negligible presence of ferromagnetic materials [32,33]. Furthermore, in vitro studies confirm that even very high-intensity static magnetic fields (SMFs) (up to 10 T) do not adversely affect cellular viability, proliferation, DNA integrity, or cell cycle progression [50,51,52,53].

Building upon this established safety profile, our study pioneers the use of a moderate-intensity SMF to induce spatial separation of immune cells (Jurkat cells) from parathyroid allografts, aiming to achieve non-immunosuppressive transplantation.

We first confirmed the safety and integrity of the parathyroid allografts under the magnetic field application. Consistent with safety data on high-intensity SMFs [50,51,52,53], moderate-intensity SMF exposure did not negatively affect the viability or proliferation of parathyroid cells in either non-encapsulated or sodium alginate-encapsulated formats (groups 1–10). Crucially, SMF exposure also did not alter the expression of key parathyroid-specific genes—VitDR, PTH, GCM2, and CaSR—which are essential for calcium homeostasis and glandular function [54,55]. Furthermore, PTH secretion levels remained stable between SMF-exposed and control groups [46,56,57,58]. These findings collectively indicate that the magnetic field application preserves the critical physiological function and genetic stability of the parathyroid graft, establishing its biocompatibility for allotransplantation.

The particularly novel component of this study was the evaluation of lymphocyte (Jurkat T cell) behavior. In the encapsulated groups (G9 and G10), lymphocytes actively accumulated around the capsule surface in the absence of SMF, suggesting active cell–cell interaction or chemotaxis. However, under SMF exposure, lymphocytes were observed to disperse away from the capsule, demonstrating that the magnetic field successfully disrupts immune cell adhesion or recruitment, which is a critical step in the immune response against the graft. This spatial separation forms the basis of our non-immunosuppressive strategy.

This phenomenon of SMF-induced altered spatial distribution highlights the dual role of encapsulation in modulating immune responses. First, the sodium alginate capsule serves as a physical barrier, preventing direct contact between lymphocytes and the allograft, which disrupts critical processes like antigen presentation and immune synapse formation. Second, the matrix may act as a biochemical filter, limiting the diffusion of immune-modulating molecules (e.g., cytokines/chemokines). In non-encapsulated systems, where direct cell–cell contact occurs freely, these fundamental immune processes may occur unchecked, potentially overriding the observed magnetic field effects. In the encapsulated system, where direct contact is prevented, the magnetic field’s influence on immune behavior is enhanced [20,21,22,23]. One plausible mechanism involves interference with lymphocyte adhesion dynamics. Cell adhesion relies on molecules like integrins (e.g., LFA-1) and their ligands (ICAM-1), which are essential for cell anchoring and migration [43]. The literature suggests that SMFs can alter membrane fluidity, lipid raft structure, and receptor clustering, all critical to adhesion stability. Furthermore, SMF may modulate cytoskeletal reorganization, particularly actin polymerization, thereby influencing cell shape and motility. These combined effects likely reduce lymphocyte adhesion to the capsule, resulting in their observed spatial separation from the transplant interface [43,44,45].

It is important to note that while this study demonstrates the functional altered spatial distribution of lymphocytes under SMF, the specific molecular pathways—such as integrin-mediated adhesion or cytoskeletal reorganization—remain hypothesized based on the established magnetobiological literature [44,45]. Direct verification of these intracellular changes was outside the scope of this functional proof-of-concept study. Future investigations using advanced microscopy to track real-time receptor clustering and actin polymerization are warranted to fully elucidate the biophysical “black box” of this partial redistribution.

Furthermore, by integrating a moderate-intensity SMF with a 3D alginate encapsulation framework, this study establishes a physiologically relevant 3D in vitro model that more accurately simulates the complex spatial dynamics and biophysical barriers of graft–host immune interactions than traditional 2D co-culture systems.

The magnetic field’s influence may extend to intracellular signaling pathways critical for immune cell migration and activation, such as PI3K-Akt, MAPK, and calcium-dependent mechanisms [43,44,45]. Disruption of these cascades through magnetic modulation could impair the lymphocytes’ ability to adhere, polarize, or respond to chemotactic cues, especially when encapsulation already restricts physical interactions.

In non-encapsulated systems, localized immune-stimulatory microenvironments (rich in paracrine signaling) may be formed, potentially overpowering the subtle modulatory effects of SMF. Encapsulation, by disrupting this direct crosstalk, allows the magnetic field to exert a more pronounced influence on lymphocyte positioning and motility.

The observed altered spatial distribution of lymphocytes is driven by magnetophoresis, a phenomenon occurring within the non-uniform magnetic field generated by the dual-magnet setup. Unlike a uniform field, the spatial gradient (∇ *B*) created by the neodymium magnets placed on the well boundaries exerts a net translational force on cells. This force arises from the difference in magnetic susceptibility between the cellular components (containing diamagnetic proteins and lipids) and the culture medium [27,59,60]. The resulting migration vector, consistently observed in our live-cell imaging, confirms that the field gradient was sufficient to overcome stochastic cellular motion and induce directed spatial separation.

Additionally, a mechanism analogous to electrotaxis—magnetically induced directional migration (magnetotaxis)—may contribute to the observed altered spatial distribution [27,59,60]. While not classically considered magnetotactic, the interplay between the SMF and cellular components containing paramagnetic ions may alter intracellular charge gradients and cytoskeletal alignment. Our observations support this hypothesis: the consistent migration of lymphocytes away from encapsulated parathyroid cells suggests the SMF acts not merely as a physical force but also as a modulator of cellular signaling and membrane polarization [39,40,41,42]. This induced migration behavior is predicted to reduce immune cell density around the graft, lowering the probability of immune activation and significantly contributing to the development of an immune-privileged transplant environment.

While these in vitro results are promising, several objective limitations must be addressed to advance this technology toward clinical application. First, the spatial geometry of the current 48-well setup constrains the maximum possible migration distance; while effective for this proof-of-concept, larger culture areas or specialized magnetic bioreactors are required to fully characterize the long-term spatial distribution of lymphocytes. Second, a significant engineering trade-off exists between magnetic field intensity and setup stability. Neodymium magnets are highly reactive and can release toxic ions—evidenced by medium discoloration and subsequent cell death—if not properly sequestered from the culture environment. To maintain biocompatibility, the current setup utilized external magnet placement, which inherently increased the distance between magnetic poles and influenced the magnetic field gradient shape. Future optimizations should explore the use of biocompatible coatings, such as medical-grade polymers or parafilm, to facilitate ‘internal’ magnet placement, thereby maximizing the field gradient and migration efficiency without compromising cell safety. Finally, the imaging-force paradox remains a technical challenge: the extreme proximity required for maximum Lorentz-force-driven altered spatial distribution often obscures the center of the well, making simultaneous high-resolution imaging difficult. Refining the transparency of insulation materials and utilizing more sophisticated magnetic configurations will be essential to overcome these barriers in future in vivo and in vitro validation studies [46,47,48,49].

While the functional outcomes of this study—specifically the preservation of PTH secretion and the induction of magnetophoretic lymphocyte exclusion—are definitive, the underlying molecular topography remains a subject for further inquiry. The directed migration of Jurkat cells likely stems from a combination of gradient-driven Kelvin forces and diamagnetic alignment of cytoskeletal polymers. However, a comprehensive transcriptomic and proteomic analysis of the ‘magneto-sensitive’ apparatus within these cells was beyond the scope of this initial validation. As the first study to propose and test this combinatorial biophysical barrier for parathyroid allotransplantation, our findings serve as a foundational proof-of-concept, establishing the parameters necessary for future mechanistic deconvolution.

Ultimately, our findings suggest that static magnetic field (SMF)-assisted encapsulation offers a highly promising, non-immunosuppressive strategy for parathyroid allotransplantation. This approach leverages two complementary protective mechanisms: physical isolation via encapsulation and the immune-modulating properties of the SMF, which collectively minimize harmful lymphocyte engagement. Together, these features may significantly enhance graft longevity and reduce rejection risk, providing a clinically viable pathway for cell-based therapies in endocrine disorders [61,62].

## 5. Conclusions

This study presents a novel, non-pharmacological strategy for parathyroid allotransplantation by integrating alginate microencapsulation with the application of a moderate-intensity static magnetic field (SMF). This approach successfully redirected lymphocyte positioning without compromising parathyroid cell viability, function, or gene expression, offering a new way to establish an immune-privileged environment. Crucially, this was achieved without the use of immunosuppressive drugs, addressing a major limitation in current protocols. This technique may pave the way for a next-generation therapeutic option for hypoparathyroidism. Future studies, including in vivo xenotransplantation and in-depth serum cytokine profiling, are critical to validate efficacy and safety and could lead to a durable, lifelong solution without the need for lifelong medication.

## Figures and Tables

**Table 1:** Basic clinical information of four parathyroid tissue donors.

Donor	Gender	Age (Years)	Diagnosis
Patient 1	Female	42	Primary hyperparathyroidism
Patient 2	Female	43	Primary hyperparathyroidism
Patient 3	Female	47	Primary hyperparathyroidism
Patient 4	Female	51	Primary hyperparathyroidism

**Table 2:** Experimental groups and culture conditions (‘’+’’ indicates presence; ‘’−‘’ indicates absence).

Group	Parathyroid Condition	Lymphocytes (Jurkat)	Magnetic Field (SMF)	Description
G1	Non-encapsulated	−	−	Control (Naked cells)
G2	Non-encapsulated	−	+	Effect of SMF on naked cells
G3	Encapsulated	−	−	Control (Encapsulated)
G4	Encapsulated	−	+	Effect of SMF on capsule
G5	(None)	+	−	Control (Lymphocytes only)
G6	(None)	+	+	Effect of SMF on Lymphocytes
G7	Non-encapsulated	+	−	Co-culture Control
G8	Non-encapsulated	+	+	Co-culture + SMF
G9	Encapsulated	+	−	Model of Rejection (Aggregated)
G10	Encapsulated	+	+	Model of Immune-Privileged Zone (Migrated)

**Table 3:** Forward (F) and reverse (R) primer sequences employed in PCR and expected amplicon lengths.

Primers		Sequence (5′ → 3′)	Product Length (bp)
PTH	F	GAGTAGAATGGCTGCGTAAGAAG	191
	R	TTCATGGCTCTCAACCAAGAC	
CaSR	F	CCAACTTGACGCTGGGATACA	221
	R	CAGCAATCGTAGAGGGAATGTG	
VitDR	F	AGCCTCAATGAGGAGCACTCCAAG	224
	R	CGGGTGAGGAGGGCTGCTGAGTA	
GCM2	F	CAAGGCACGGCTGAAACAG	295
	R	GCCCTCGACAAGGAATCAACT	
IL-2	F	TACAAGAACCCGAAACTGACTCG	194
	R	ACATGAAGGTAGTCTCACTGCC	
IL-1α	F	AGATGCCTGAGATACCCAAAACC	147
	R	CCAAGCACACCCAGTAGTCT	
IL-1β	F	AGCTACGAATCTCCGACCAC	189
	R	CGTTATCCCATGTGTCGAAGAA	
B-Actin	F	CATGTACGTTGCTATCCAGGC	142
	R	CTCCTTAATGTCACGCACGAT	

## Data Availability

The original contributions presented in this study are included in the article/Supplementary Materials. Further inquiries can be directed to the corresponding author.

## Supplementary Materials

The following supporting information can be downloaded at: https://www.mdpi.com/article/10.3390/cimb48040388/s1.

## Abbreviations

The following abbreviations are used in this manuscript:

µg	Microgram
µL	Microliter
µm	Micrometer
µT	Microtesla
BSA	Bovine Serum Albumin
Ca	Calcium
CaCl_2_	Calcium Chloride
CaSR	Calcium-Sensing Receptor
cDNA	Complementary/Copy Deoxyribonucleic acid
CO_2_	Carbon Dioxide
DC	Direct Electric Current
DMEM-HG	Dulbecco’s Modified Eagle Medium-High Glucose
DNA	Deoxyribonucleic Acid
ELISA	Enzyme-Linked Immunosorbent Assay
EtHD-1	Ethidium Homodimer-1
FBS	Fetal Bovine Serum
G	Gauss
GCM2	Glial Cells Missing Homolog 2
ICAM-1	The Intercellular Adhesion Molecule-1
ICNIRP	The International Commission on Non-Ionizing Radiation Protection
IL	Interleukin
LFA-1	Lymphocyte Function-Associated Antigen-1
MAPK	Mitogen-Activated Protein Kinase
MFs	Magnetic Fields
mg	Milligram
mL	Milliliter
MRI	Magnetic Resonance Imaging
mT	Millitesla
MTS	Proliferation Assay Reagent
n	Sample Size
nm	Nanometer
OD	Optical Density
P/S	Penicillin/Streptomycin
p	*p*-value (Statistical Significance)
PBS	Phosphate Buffered Saline
PCR	Polymerase Chain Reaction
pg	Picogram
PI3K-Akt	Phosphoinositide 3-kinase-Protein Kinase B
PTH	Parathyroid Hormone
PVDF	Polyvinylidene Fluoride
RPM	Revolutions per Minute
RPMI	Roswell Park Memorial Institute Medium
SI	International System of Units
SMF	Static Magnetic Field
T	Tesla
VitDR	Vitamin D Receptor
