# Supplementary material for: Development of a Novel Immunoprotective Culture System for Parathyroid Allografts: Utilizing Static Magnetic Fields to Modulate Lymphocyte Migration
Source: Curr Issues Mol Biol. 2026 Apr 10;48(4):388. doi: 10.3390/cimb48040388 (PMC13114947; doi:10.3390/cimb48040388)
Supplement: Supplementary file 1 [file cimb-48-00388-s001.zip › cimb-4210981-caption.docx]

**Video S1:** Live-cell video of Group 9 samples (encapsulated parathyroid cells, co-cultured with Jurkat cells) (without static magnetic field) after 24 (**A**), 48 (**B**), and 72 (**C**) hours, magnification: X 2.5.;

**Video S2:** Live-cell video of Group 10 samples (encapsulated parathyroid cells, co-cultured with Jurkat cells) (with static magnetic field) after 24 (**A**), 48 (**B**), and 72 (**C**) hours, magnification: X 2.5.
